# Targeted Detection of 76 Carnitine Indicators Combined with a Machine Learning Algorithm Based on HPLC-MS/MS in the Diagnosis of Rheumatoid Arthritis

**DOI:** 10.3390/metabo15030205

**Published:** 2025-03-18

**Authors:** Rui Zhang, Juan Wang, Xiaonan Zhai, Yuanbing Guo, Lei Zhou, Xiaoyan Hao, Liu Yang, Ruiqing Xing, Juanjuan Hu, Jiawei Gao, Fengjuan Wang, Jun Yang, Jiayun Liu

**Affiliations:** 1 Department of Clinical Laboratory Medicine, Xijing Hospital, Fourth Military Medical University, 127 Changle West Road, Xi’an 710032, China; ruizhangzr@fmmu.edu.cn (R.Z.); wangju1218@fmmu.edu.cn (J.W.); xiaonanzhaizxn@fmmu.edu.cn (X.Z.); yuanbingguo@fmmu.edu.cn (Y.G.); zhoulei1985@fmmu.edu.cn (L.Z.); haoxiaoyan@fmmu.edu.cn (X.H.); zhangzh@fmmu.edu.cn (L.Y.); xrqing210b@fmmu.edu.cn (R.X.); hujuanjuanhjj@fmmu.edu.cn (J.H.); gaojiawei1314@fmmu.edu.cn (J.G.); wangfengjuan@fmmu.edu.cn (F.W.); 2 Comprehensive Cancer Center, Department of Entomology and Nematology, University of California, Davis, CA 95616, USA

**Keywords:** rheumatoid arthritis diagnosis, carnitine metabolism, HPLC-MS/MS analysis, machine learning diagnostic models, inflammatory biomarkers, autoimmune disease pathogenesis

## Abstract

Background/Objectives: Early diagnosis and treatment of rheumatoid arthritis (RA) are essential to reducing disability. However, the diagnostic criteria remain unclear, relying on clinical symptoms and blood markers. Methods: Using high-performance liquid chromatography–mass spectrometry (HPLC-MS/MS) targeted detection, we evaluated 76 carnitine indicators (55 carnitines and 21 corresponding ratios) in the serum of patients with RA to investigate the role of carnitine in RA. A total of 359 patients (207 patients with RA and 152 healthy controls) were included in the study. Screening involved three methods and integrated 76 carnitine indicators and 128 clinical indicators to identify candidate markers to establish a theoretical basis for RA diagnosis and new therapeutic targets. The diagnostic model derived from the screened markers was validated using three machine learning algorithms. Results: The model was refined using eight candidate indicators (C0, C10:1, LYMPH, platelet distribution width, anti-keratin antibody, glucose, urobilinogen, and erythrocyte sedimentation rate (ESR)). The receiver operating characteristic curve, sensitivity, specificity, and accuracy of the V8 model obtained from the training set were >0.948, 79.46%, 92.99%, and 89.18%, whereas those of the test set were >0.925, 78.89%, 89.22%, and 85.87%, respectively. Twenty-four carnitines were identified as risk factors of RA, with three significantly correlating with ESR, four with anti-cyclic citrullinated peptide antibody activity, two with C-reactive protein, five with immunoglobulin-G, eight with immunoglobulin-A levels, and eleven with immunoglobulin-M levels. Conclusions: Carnitine is integral in the progression of RA. The diagnostic model developed shows excellent diagnostic capacity, improving early detection and enabling timely intervention to minimize disability associated with RA.

## 1. Introduction

Rheumatoid arthritis (RA) is a chronic inflammatory joint disease that manifests symmetrically and multiarticularly, with a high rate of disability [1]. It is a major cause of labor loss and disability worldwide, with a global incidence of approximately 0.5–1% and a prevalence of approximately 0.32–0.38% in China [2]. Its etiology may be linked to susceptible genes, epigenetic modifications, and environmental factors [3]. Although the production of autoantibodies is a major factor in the development of RA and other autoimmune diseases, the underlying mechanisms of the initial autoimmune response and key biological pathways driving it remain unclear [4]. In addition, some patients with RA test positive for antibodies, while others do not, and the differences in the pathogenesis of these two types of RA remain unclear [5]. Metabolomic technology has received extensive attention in studying the occurrence and development of RA, revealing metabolic disorders involved in its pathogenesis and progression [6]. These abnormalities affect several metabolic pathways, directly or indirectly promoting inflammatory and immune responses during the pathogenesis of RA [7]. Plasma metabolite concentrations, including carnitine, are associated with disease activity in RA [8]. Some carnitine metabolic pathways are key regulators of the adaptive immune responses to inflammation [9]. Although global advances in metabolomics have provided theoretical insights into the pathogenesis and diagnosis of RA, the results have been inconsistent owing to differences in influencing factors [10]. While several metabolic pathways are linked with RA, their exact trends remain uncertain, highlighting the need for targeted research on changes in carnitine levels in RA.

Studies have identified a positive association between delayed diagnosis of RA and the degree of joint injury [11]. Additionally, delayed diagnosis exceeding five or six months increases the risk of joint erosion, while delayed diagnosis exceeding 12 months could cause severe and substantial joint injury [12]. Therefore, early diagnosis and treatment of RA are essential to alleviating disability. However, current RA diagnosis still relies on clinical symptoms and laboratory markers such as anti-cyclic citrullinated peptide antibody (anti-CCP) and rheumatoid factor (RF), which are not specific to RA and are generally elevated in older adults [13,14,15]. The high misdiagnosis rate in seronegative patients with RA and a limited number of validated biomarkers significantly impede the accurate diagnosis, prognosis, and risk prediction of RA.

Machine learning algorithms are becoming increasingly pertinent in disease diagnosis by integrating data from computer science, statistics, and medicine [16]. Machine learning can improve the statistical ability and effectiveness of simple and efficient diagnostic models for clinicians to guide disease diagnosis [17]. Some researchers have proposed that carnitine could be used as a potential biomarker for auxiliary diagnosis, drug therapy monitoring, and efficacy evaluation of RA [18,19]. The integration of multiple metabolites will substantially improve the sensitivity and specificity for predicting RA [20]. However, very few studies have systematically investigated multiple machine learning algorithms combined with carnitine levels and clinical indicators for the diagnosis of RA. The use of multiple methods to identify metabolic biomarkers that can predict RA is also crucial to establish a theoretical basis for the diagnosis of RA and identify new therapeutic targets.

Based on significant changes in carnitine levels in patients with RA, we employed HPLC-MS/MS technology to target 76 carnitine indicators (55 carnitines and 21 corresponding ratios) combined with 128 clinical indicators, adopting multiple methods to screen candidate markers for the diagnostic model. We established and validated the model using three machine learning algorithms. Correlation and risk factor analyses were performed to investigate the role of carnitine in the development and occurrence of RA.

## 2. Materials and Methods

### 2.1. Participants

A total of 207 patients with RA were recruited from Xijing Hospital between 1 July 2023, and 1 December 2024, meeting the criteria of the American College of Rheumatology 1987 [21] and 2010 [22]. A control group of healthy individuals (n = 152) was also recruited. All patients with RA did not receive any prior treatment, and the exclusion criteria were as follows: (1) prior immunosuppressive therapy or a history of immunosuppressive therapy, (2) prior hormone therapy or a history of hormone therapy, (3) prior chemotherapy and radiotherapy or a history of chemotherapy and radiotherapy, (4) current antibiotic use, and (5) data deletion rate >70%. The study protocol was approved by the Ethics Committee of Xijing Hospital (KY20212027-C-1), and the written informed consent of all participants in the study was obtained.

### 2.2. Blood Preparation

Patients fasted for more than eight hours, and venous blood was drawn by an experienced nurse in the early morning.

### 2.3. Data Collection

All carnitine and laboratory tests were performed at the clinical laboratory of Xijing Hospital. Quality control measures were implemented before, during, and after testing to ensure accuracy. Clinical test results were stored in a laboratory information system. Patients’ basic clinical information was recorded and entered by trained personnel.

### 2.4. HPLC-MS/MS Materials and Equipment

HPLC-MS/MS was used to analyze 55 carnitines [free carnitine (C0), acetylcarnitine (C2), propionylcarnitine (C3), butyrylcarnitine (C4), isovalerylcarnitine (C5), caproylcarnitine (C6), heptanylcarnitine (C7), octanoylcarnitine (C8), nonylcarnitine (C9), decanoylcarnitine (C10), dodecanoylcarnitine (C12), tetradecanoylcarnitine (C14), cetacylcarnitine (C16), heptadecanoylcarnitine (C17), octadecanoylcarnitine (C18), eicosanoylcarnitine (C20), docosenoylcarnitine (C22), tetracosanoylcarnitine (C24), pentacanoyl-carnitine (C25), pentacosanoyl-carnitine(C26), malonylcarnitine (C3DC), 3-hydroxy butyrylcarnitine (C4OH), methylmalonylcarnitine (C4DC), 3-hydroxy isovalerylcarnitine (C5OH), tiglylcarnitine (C5:1), glutarylcarnitine (C5DC), hexanoylcarnitine (C6OH), adipylcarnitine (C6DC), octenoylcarnitine (C8:1), decenoylcarnitine (C10:1), decadienoylcarnitine (C10:2), dodecenoylcarnitine (C12:1), myristoleylcarnitine (C14:1), tetradecadienoylcarnitine (C14:2), 3-hydroxy myristoylcarnitine (C14OH), hexadecenoylcarnitine (C16:1), 3-hydroxy palmitoylcarnitine (C16OH), 3-hydroxy palmitoleylcarnitine (C16:1OH), octadecenoylcarnitine (C18:1), linoleylcarnitine (C18:2), 3-hydroxy octadecanoylcarnitine (C18OH), 3-hydroxy octadecenoylcarnitine (C18:1OH), eicosenoylcarnitine (C20:1), decatrienoylcarnitine (C10:3), eicosadienoylcarnitine (C20:2), eicosatrienoylcarnitine (C20:3), octylodiacylcarnitine (C8DC), sebacylcarnitine (C10DC), dodecanodiacylcarnitine (C12DC), tetradecanodiacylcarnitine (C14DC), hexadecanodiacylcarnitine (C16DC), octadecanodiacylcarnitine (C18DC), eicosanodiacylcarnitine (C20DC), hydroxydodecanoyl carnitine (C12-OH), hydroxy-eicoicylcarnitine (C20-OH)] and 21 corresponding ratios (C0/C2, C0/C16, C3/C0, C3/C2, C3/C16, C4/C3, C5/C4, C5DC/C8, C5DC/C16, C8/C2, C8/C3, C8/C10, C8/C12, C8/C16, C14:1/C16, C16OH/C16, C24/C22, C25/C22, C26/C20, C26/C22, C26/C24). An internal standard included free carnitine internal standard (d9-C0), acetylcarnitine internal standard (d3-C2), propionylcarnitine internal standard (d3-C3), butyrylcarnitine internal standard (d3-C4), isovalerylcarnitine internal standard (d9-C5), octanoylcarnitine internal standard (d3-C8), tetradecanoylcarnitine internal standard (d9-C14), and cetacylcarnitine internal standard (d3-C16) (Cambridge Equivalent Chemistry Laboratory) dissolved in acetonitrile as an extraction reagent.

### 2.5. HPLC-MS/MS Sample Pretreatment

To detect the 76 carnitine indicators, 50 μL of serum was dropped on a blank blood collection filter paper and allowed to permeate. A circular disc with a 3.5 mm diameter of the dry serum filter paper was accurately cut and extracted with 100 μL of extraction reagent at 25 °C for 15 min. All the extract was removed into a new tube and blown dry with nitrogen. A derivatization reagent was prepared by n-butanol and acetyl chloride in a 9:1 volume ratio. Then, 60 μL of the derivatization reagent was added to the dry sample, and the reaction was allowed to proceed for 20 min at 65 °C. The final derivatized solution was dried under nitrogen and re-dissolved in 100 μL of acetonitrile. This was used as the detection solution for mass spectrum analysis.

### 2.6. Mass Spectrum Analysis

Acetonitrile (100%) was used as the mobile phase for HPLC (HPLC 1100, Agilent, Waldbronn, Germany) to detect the 76 carnitine indicators (55 carnitines and 21 corresponding ratios). The HPLC parameters are shown in Table S2. The sample injection volume for mass spectrometry (3200 QTRAP, AB Sciex, Darmstadt, Germany) detection was 20 μL. The experiments were performed with an electrospray ionization source by precursor scan type (85 Da, 210.00 Da–600.00 Da) in positive ion mode, and the optimized parameters are shown in Table S3. The entrance started from 15.27 v, and stopped at 27.48 v. Collision energy started from 35.00 v, and stopped at 45.00 v.

### 2.7. Statistical Analysis

The mass spectrum data were analyzed using Analyst (version 1.6.2) and ChemView software (version 1.6.1; AB Sciex, Darmstadt, Germany). SPSS (version 23.0; IBM, Armonk, NY, USA) was utilized for group analysis of the quantitative data, which were compared using independent *t*-test analysis and *p*-values (statistical significance set at *p* < 0.05). Quantitative data were expressed as mean ± standard deviation. Data visualization was carried out using GraphPad Prism (version 5; GraphPad Software, San Diego, CA, USA) and R software (version 3.6.2; R Statistical Computing Project; statistical significance at *p* < 0.05). The orthogonal partial least squares discriminant analysis (OPLS-DA) model was used to evaluate the differences between the RA and control groups and the metabolic similarities within the groups. The diagnostic efficiency of the model was evaluated by the receiver operating characteristic (ROC) curve. The small molecule pathway database (SMPDB) was used to illustrate the pathways of differentially expressed carnitine. Binary logistic regression analysis was used to assess carnitine levels and their ratio as risk factors for RA.

### 2.8. Establishment of the Diagnostic Models

To establish an effective diagnostic model, 30 indicators with the highest contribution values were screened using recursive feature elimination logistic regression, mutual information, and spearman methods. Common indicators were used to build the model. To enhance the validity of the RA diagnostic model, three machine learning classification algorithms (logistic regression (LR), Gaussian Bayes (GNB), and xgBoost) were used to construct and validate the model. Performance indices such as sensitivity, specificity, accuracy, positive predictive value, negative predictive value, area under the curve (AUC), and ROC curves were evaluated. To ensure the stability of the algorithms, the data were cross-validated 10 times, with each iteration comprising nine training sets and one test set.

### 2.9. SHAP (SHapley Additive exPlanations) Interpretation

In the application of diagnostic models, simple models (e.g., linear models) are easier to interpret, but they are not as accurate as complex models [23]. The availability of big data makes it feasible to use complex models for disease diagnosis, but how to balance the accuracy and interpretability of model outputs remains to be investigated [24]. We use the SHAP model to explain the diagnostic model we built. The SHAP model is characterized by SHAP values that illuminate the decision-making process of a machine learning model by evaluating importance as a unifying metric [25,26]. The SHAP values provide a unique metric of the importance of additional features, combining Shapley regression, Shapley sampling, and imputation of quantitative inputs to influence the features to ensure that the contribution of each feature’s contribution to model predictions is accurately quantified and assigned [27]. By converting the predicted values to the sum of these attributes, SHAP helps to provide a deeper understanding of the model’s behavior, highlighting which features are most influential in driving predictions and providing insights into the dynamics behind the model’s predictions [28,29].

## 3. Results

### 3.1. Study Participants

Table S1 summarizes the basic details of the participants, including height, weight, blood pressure, lifestyle habits, expression levels, and differences in all measured indicators. The RA and control groups had similar basic physiological traits, but differences in living habits were observed, which could be attributed to the impact of RA on the patients’ quality of life, which could not be ignored. Compared with the control group, there were statistically significant differences in 22 carnitines and their ratios in the RA group (13 indicators were significantly reduced and 9 indicators were significantly increased), alongside 73 clinical indicators.

### 3.2. Distribution and Metabolic Pathway of Each Measurement Index

**Figure 1:**
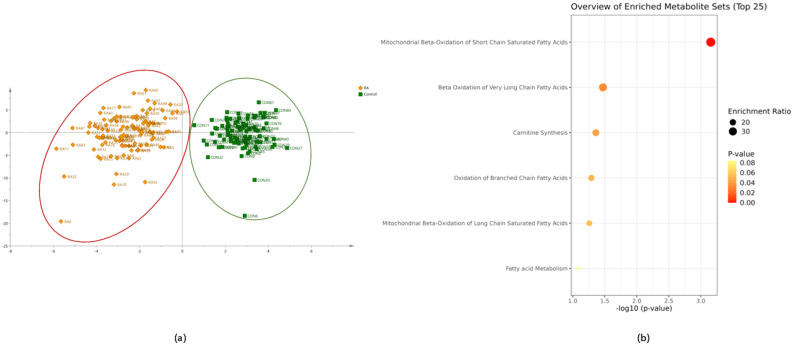
(**a**) Orthogonal partial least squares discriminant analysis (OPLS-DA) model comparing the control group (green) and rheumatoid arthritis group (orange). (**b**) The changes in the metabolic pathway differed significantly between the control and the RA groups analyzed by SMPDB.

The results of the OPLS-DA model revealed overall differences across all indicators between the RA and control groups (Figure 1a). The permutation test validation model confirmed that the model could effectively differentiate between the groups.

### 3.3. Metabolic Pathways

By enrichment pathway analyses, differential metabolic pathways and corresponding carnitine with statistically significant differences were identified (importance of predictors > 1.0). Different pathways in the SMPDB of the RA and control groups were compared. Mitochondrial beta-oxidation of short-chain saturated fatty acids, beta-oxidation of very long-chain fatty acids, carnitine synthesis, oxidation of branched-chain fatty acids, mitochondrial beta-oxidation of long-chain saturated fatty acids, and fatty acid metabolism were the pathways with the highest degrees of differentiation (Figure 1b).

### 3.4. Performance Evaluation of Candidate Indicators for Models Based on Classification Algorithms

**Figure 2:**
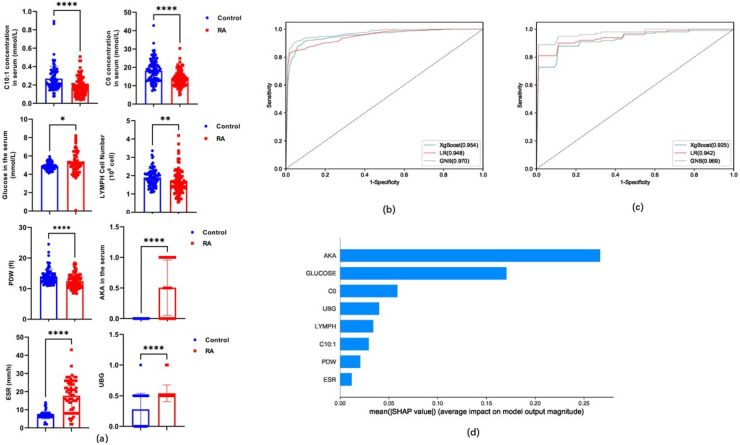
(**a**) Levels of free carnitine (C0), decenoylcarnitine (C10:1), lymphocyte count (LYMPH), platelet distribution width (PDW), anti-keratin antibody (AKA), glucose, urobilinogen (UBG), and erythrocyte sedimentation rate (ESR) in RA and control groups. (**b**) Receiver Operating Characteristic (ROC) curve of the V8 model derived from the rheumatoid arthritis (RA) diagnostic training set. (**c**) ROC curve of V8 model obtained from RA diagnostic validation set. (**d**) SHapley Additive exPlanation (SHAP) model for eight indicators of the V8 model. *, *p* < 0.05; **, *p* < 0.01; ****, *p* < 0.0001.

Based on the differences in the expression of carnitine and clinical indicators, 30 indicators with the greatest differences were cross-screened using three methods (recursive feature elimination logistic regression, mutual information, and spearman). These common indicators were then used as potential common biomarkers for modeling the RA and control groups. Eight markers (C0, C10:1, lymphocyte count [LYMPH], platelet distribution width [PDW], anti-keratin antibody [AKA], glucose, urobilinogen [UBG], and erythrocyte sedimentation rate [ESR]) were the common indicators cross-extracted by these three methods and selected as candidate markers to establish a diagnostic model for RA (V8) (*p* < 0.0001) (Figure 2a). The validity of the diagnostic model based on the eight candidate indices was established and verified using three classifier algorithms. The ROC, sensitivity, specificity, and accuracy of the V8 model obtained from the training set were >0.948, 79.46%, 92.99%, and 89.18%, respectively (Table 1 and Figure 2b). The ROC curve, sensitivity, specificity, and accuracy of the V8 model validated using the test set were >0.925, 78.89%, 89.22%, and 85.87%, respectively (Table 1 and Figure 2c). The SHAP model of the eight indicators in the V8 model revealed that AKA contributed the most, followed by glucose, C0, UBG, LYMPH, C10:1, PDW, and ESR (Figure 2d).

**Figure 3:**
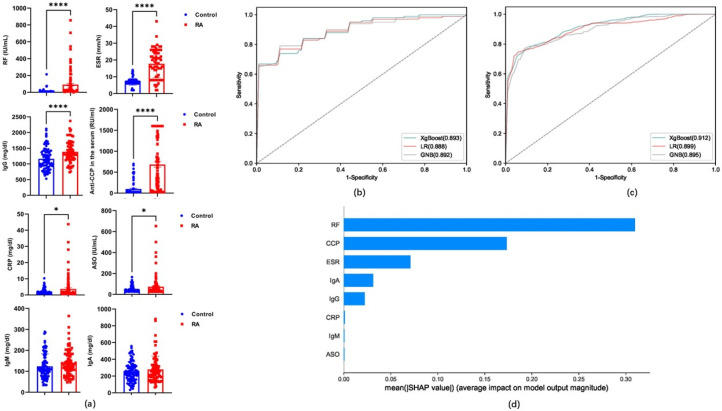
(**a**) Levels of ESR, anti-cyclic citrullinated peptide antibody (anti-CCP), anti-streptolysin O (ASO), rheumatoid factor (RF), C-reactive protein (CRP), immunoglobulin-G (IgG), immunoglobulins-A (IgA), and immunoglobulins-M (IgM) in RA and control groups. (**b**) ROC curve of the VR8 model from the RA diagnostic training set. (**c**) ROC curve of VR8 model obtained from RA diagnostic validation set. (**d**) SHAP model for eight indicators of the VR8 model. *, *p* < 0.05; ****, *p* < 0.0001.

To investigate the feasibility of the RA diagnostic model, we employed eight rheumatoid-specific clinical measures (ESR, anti-CCP, anti-streptolysin O [ASO], RF, C-reactive protein [CRP], immunoglobulin-G [IgG], immunoglobulins-A [IgA], and immunoglobulins-M [IgM]) to establish a diagnostic model (VR8) (Figure 3a). Three classification algorithms were used to establish and evaluate the performance of the VR8 model using these indicators to diagnose RA. The ROC, sensitivity, specificity, and accuracy of the VR8 model established using the training set were >0.895, 72.73, 85.14, and 81.97%, respectively (Table 1 and Figure 3b). Similarly, in the test set of the VR8 model, the values were >0.888, 73.89%, 82.64%, and 79.00%, respectively (Table 1 and Figure 3c). The SHAP model of the eight indicators in the VR8 model revealed that RF contributed the most, followed by anti-CCP, ESR, IgA, IgG, CRP, IgM, and ASO (Figure 3d).

**Figure 4:**
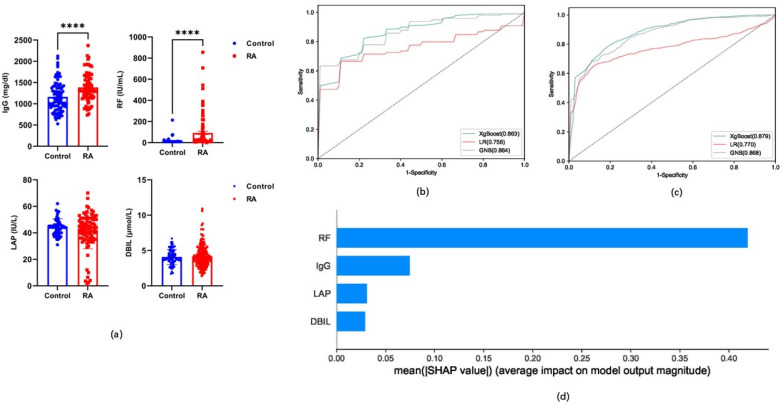
(**a**) Levels of direct bilirubin‌‌ (DBIL), leucine aminopeptidase (LAP), RF, and IgG in RA and control groups. (**b**) ROC curve of the V4 model derived from the RA diagnostic training set. (**c**) ROC curve of V4 model obtained from RA diagnostic validation set. (**d**) SHAP model highlighting eight indicators of the V4 model. ****, *p* < 0.0001.

To further investigate the role of carnitine in RA diagnosis, we established an effective diagnostic model by integrating eight rheumatoid-specific and common clinical indicators. The 30 indicators with the greatest differences were cross-screened using the three methods, and potential markers were extracted to model the RA and control groups. Four indicators (direct bilirubin‌‌ [DBIL], leucine aminopeptidase [LAP], RF, and IgG) were selected as candidates for establishing the RA diagnostic model (V4) (*p* < 0.0001) (Figure 4a). Three classification algorithms were used to test the validity of the diagnostic V4 model built using four candidate indicators. The ROC, sensitivity, specificity, and accuracy obtained in the V4 model established using the training set were >0.770, 56.90%, 77.89%, and 74.34%, respectively (Table 1, Figure 4b). Moreover, these values in the test set of the V4 model were >0.756, 55.56%, 68.67%, and 72.32%, respectively (Table 1 and Figure 4c). The SHAP model of the four indicators in the V4 model showed that RF contributed the most, followed by IgG, LAP, and DBIL (Figure 4d).

**Figure 5:**
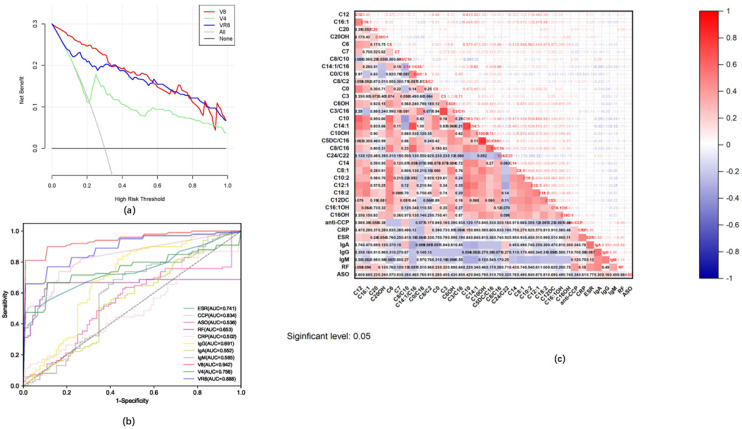
(**a**) Non-trend correspondence analysis charts for V8, V4, and VR8 models. (**b**) Model ROC curves obtained from V8 and VR8 models: ESR, anti-CCP, ASO, RF, CRP, IgG, IgA, and IgM for RA and control groups. (**c**) Correlation graph of carnitine in RA with clinical indicators.

The analysis results of the decision curves for V8, VR8, and V4 show that the net benefit of the V8 diagnostic model was higher than that of the VR8 and V4 diagnostic models, especially when the high-risk threshold was <0.3 (Figure 5a). The ROC comparison of V8, VR8, V4, ESR, anti-CCP, ASO, RF, CRP, IgG, IgA, and IgM (Figure 5b) showed that the AUCs of V8, VR8, V4, and the clinically used rheumatoid-specific markers were >0.942, 0.888, 0.756, and 0.502, respectively.

### 3.5. The Role of Carnitine and Its Ratio in RA

To explore the potential value of carnitine and its ratios in RA, binary logistic regression modeling was performed to identify the indicators that predicted the occurrence of RA (Table S4). Twenty-four carnitines and their ratios were identified as risk factors for RA. Elevated levels of C2, C7, C14, C22, C25, C8CD, C5CD/C8, C8/C10, C141/C16, C16OH/C16, and C25/C22 were associated with an increased risk of RA. Elevated levels of C0, C4, C6, C8, C10, C24, C101, C142, C10OH, C4/C3, C5CD/C16, C8/C2, and C8/C16 were negatively associated with RA development.

ESR, anti-CCP, ASO, RF, CRP, IgG, IgA, and IgM levels were closely related to the occurrence and development of RA. Pearson correlation coefficients and *p*-values were used to evaluate the association between carnitine and RA-related clinical indicators to explore the role of carnitine in the pathogenesis and progression of RA (Figure 5c). Pearson correlation coefficient analysis showed that three carnitines were significantly correlated with ESR in the RA disease group (C12, C16:1, and C20OH). Four carnitines and their ratios were significantly correlated with anti-CCP activity (C6, C7, C8/C10, and C14:1/C16). Two carnitines and their ratios were significantly correlated with CRP levels (C0/C16 and C8/C2). Five carnitines and their ratios were significantly correlated with IgG (C0, C3, C6OH, C3/C16, and C14:1/C16). Eight carnitines and their ratios were significantly correlated with IgA levels (C10, C14:1, C10OH, C5C/C16, C8/C10, C8/C16, C14:1/C16, and C24/C22). The ratios of 11 carnitines were significantly correlated with IgM levels (C10, C14, C8:1, C10:2, C12:1, C14:1, C18:2, C12C, C16:1OH, C16OH, and C14:1/C16).

## 4. Discussion

In rheumatoid arthritis, metabolic disorders can increase immune cell invasiveness, disease activity, and disability [30]. Previous studies evaluating plasma metabolism in high-risk individuals or patients with rheumatoid joint disease have linked significant changes in carnitine and choline concentrations to the future development or presence of RA [31]. However, many metabolic studies lack targeting, thus limiting the accuracy [32,33]. Additionally, due to regional or patient differences, carnitine trends in RA are inconsistent [30,34]. Currently, there are no clear diagnostic criteria for RA, and linking metabolic disorders with clinical indicators to improve diagnosis remains an area for further study [35]. Therefore, this study aimed to clarify the changing trends and roles of 76 carnitine indicators (55 carnitines and 21 corresponding ratios) in inflammatory arthritis, establishing a diagnostic model for RA to aid early warning and minimize disability through timely intervention.

Our study identified changes in serum levels of multiple carnitines in patients with RA compared to controls, suggesting disturbed carnitine metabolism in RA, consistent with previous findings reported in the literature [36]. Serum C8/C10, C14:1/C16, and C25/C22 levels were significantly elevated in patients with RA, corresponding with an increased risk of RA and positively associated with RA development. Elevated levels of C0, C4, C6, C8, C10:1, C4/C3, and C8/C10 were associated with a reduced risk of RA, and these elevated levels inversely correlated with risk factors for RA. However, these markers were significantly reduced in the serum of patients with RA. Our results confirm that a disorder in carnitine metabolism is related to the pathogenesis of RA. Additionally, the correlation with clinical indicators related to RA suggests that multiple carnitines are closely related to the formation of infection, immunity, and antibody complexes in patients with RA. Thus, this provides evidence that carnitine is closely related to the development of RA, but the specific mechanism remains an area for further research. Compared to the change in long-chain carnitine concentration, the change in short-chain carnitine concentration may play a more important role in the occurrence and development of RA. Su et al. analyzed the plasma of patients with RA using non-targeted metabolomics and identified three metabolites, namely 4-acetamidobutanoate, C5 carnitine, and C5:1 carnitine, that were significantly associated with the incidence of serotype RA, although no significant association was found after adjustment [37], supporting our conclusion.

In this study, we observed that carnitine plays a role in the onset and development of RA following targeted analysis of multiple serum carnitines in patients with RA. C0 is a naturally occurring compound in most body tissues that transports long-chain fatty acid acyl-CoA into the mitochondria for beta-oxidation [38]. It is synthesized by methionine and lysine [39]. C0 protects mitochondrial function through multiple mechanisms, such as inhibiting the production of reactive oxygen species, which helps in preventing oxidative stress, inflammation, and apoptosis [40,41]. Consequently, it holds promise for the treatment of many diseases, such as weight loss, angina, and heart failure [42,43]. In this study, C0 was a candidate diagnostic marker screened by the three methods, and it proved to be one of the diagnostic markers with the largest contribution to the diagnostic model. C0 is one of the markers exhibiting the largest concentration change in RA serum and is also significantly correlated with CRP and IgG. This indicates that the serum concentration of C0 plays a key role in the occurrence and development of RA, but the specific mechanism remains unexplored. Additionally, as a safe drug already in clinical use, C0 plays a positive role in the treatment of RA. Pawlik et al. [19] and Maeda et al. [44] reported that the expression of organic cation/carnitine transporter protein 1 (OCTN1, SLC22A4) was regulated by RUNX1, inflammatory cytokines, and NF-κB, and that treatment with C0 might affect the associated inflammatory pathways; Katturajan et al. [45] reported that supplementation with L-carnitine and zinc prevented damage to the intestinal tract in rats with methotrexate arthritis by restoring enterocyte proliferation and micronutrients to prevent intestinal damage in methotrexate-treated rats with adjuvant arthritis. Therefore, the specific mechanism by which C0 can alleviate the symptoms of RA patients still needs further research. Moreover, the ratio of carnitine C8/C10 is another crucial indicator. In our study, it is not only one of the risk factors for RA but also correlated with various RA-related clinical indicators, suggesting that the imbalance of C8 and C10 plays a significant role in the occurrence and development of RA. All these results suggest that the disturbance of carnitine may play an important role in the pathogenesis of RA patients.

Currently, the diagnostic efficiency of commonly used RA-specific laboratory diagnostic markers is low, with an AUC of approximately 0.6. Therefore, many studies have developed new RA diagnostic models [46]. Luan et al. selected 26 serum markers from non-targeted metabolomic analyses to build a machine learning-based predictive model that can help diagnose seronegative patients with RA [18]. The accuracy of the model based on the binary logistic regression algorithm was 100% (AUC = 1.00) after cross-validation. The model was tested using an independent validation set (n = 82) with an AUC of 0.91, a sensitivity of 89.7%, and a specificity of 90.6%. Although the diagnostic capability is relatively high, the inclusion of too many unusual clinical indicators reduces clinical practicability. Similarly, Zhu et al. utilized gut microbes, plasma metabolite profiles, gene expression profiles, and protein and phosphoprotein profiles to study the diagnostic markers of RA [47]. These were subsequently screened by multiple omics, and four metabolite biomarkers (1-palmitoyl-sn-glycero-3-phosphocholine, hexadecanedioic acid, L−tryptophan, N6−methyl−L−lysine) were obtained, with diagnostic performance generally above 0.7 (AUC) alone and a combined AUC as high as 0.958 identified. However, the model and marker level changes in this study have not been validated. In our study, three methods were used to screen 76 carnitine indicators (55 carnitines and 21 corresponding ratios) and 128 clinical indicators. Additionally, three machine learning algorithms were employed to establish and validate the diagnostic model for RA, including (1) a combined diagnosis using eight RA-specific markers, (2) a model integrating carnitine and clinical indicators, including RA-specific markers, and (3) a detailed diagnosis combining RA-specific markers and other various clinical indicators. The three diagnostic models developed in this study can be applied to RA diagnosis, all achieving AUC values higher than those of single RA-specific indicators. Upon validation, the diagnostic efficiency of the V8 model was found to be superior to that of the VR8 and V4 models in terms of AUC, sensitivity, specificity, and accuracy. It also surpassed various newly developed RA diagnostic models, establishing it as the most effective. By including both antibody-positive and antibody-negative patients with RA and utilizing common clinical markers and carnitine changes, our model addresses shortcomings in antibody diagnosis.

This study has some limitations. First, all participants were recruited from a single center, which may have introduced selection bias. Second, our RA diagnostic model requires validation in larger and more diverse populations to ensure applicability and reliability.

## 5. Conclusions

In this study, using HPLC-MS targeted detection of 76 carnitine indicators (55 carnitines and 21 corresponding ratios) in the serum of patients with RA combined with **128** clinical indicators demonstrated carnitine’s key role in the occurrence and development of RA. Three methods were used to identify candidate markers from over 100 indicators, and three machine learning algorithms established the RA diagnostic model of the screened markers (eight RA-specific markers, combined diagnosis of eight kinds of carnitine and clinical indicators, including RA-specific markers, and combined diagnosis of various clinical indicators). The correlation we found between carnitine and RA-related indicators can provide more possibilities and options for future overall treatment strategies or personalized medical regimens for RA in the clinic, as well as provide a database for RA-related mechanism studies. The V8 diagnostic model was found to be the best for RA. Our diagnostic model incorporates antibody-positive and antibody-negative patients with RA and uses common clinical markers alongside carnitine changes to compensate for the deficiencies in antibody diagnosis. Therefore, it can contribute to the early detection of RA and minimize disability through early intervention.

## Figures and Tables

**Table 1:** Evaluation of the model for RA and control.

	Algorithms		Sensitivity	Specificity	Accuracy	PPV	NPV	MCC	AUC
V8	xgBoost	Training set	88.10%	92.99%	90.46%	93.14%	87.95%	81.09%	0.954 (0.924–0.984)
		Test set	82.89%	89.22%	85.87%	90.14%	83.92%	73.06%	0.925 (0.886–0.964)
	LR	Training set	84.63%	95.29%	89.76%	95.09%	85.21%	80.11%	0.948 (0.916–0.98)
		Test set	84.00%	93.33%	88.47%	93.96%	85.28%	78.27%	0.942 (0.908–0.976)
	GNB	Training set	79.46%	99.64%	89.18%	99.59%	81.87%	80.27%	0.970 (0.946–0.994)
		Test set	78.89%	98.89%	88.50%	99.09%	82.36%	79.57%	0.969 (0.944–0.994)
VR8	xgBoost	Training set	79.01%	85.14%	81.97%	85.18%	79.05%	64.19%	0.912 (0.87–0.954)
		Test set	75.89%	82.67%	79.08%	82.58%	76.88%	59.00%	0.912 (0.87–0.954)
	LR	Training set	77.55%	86.84%	82.02%	86.40%	78.27%	64.53%	0.899 (0.854–0.944)
		Test set	76.00%	82.89%	79.00%	84.21%	77.46%	60.23%	0.888 (0.841–0.935)
	GNB	Training set	72.73%	92.63%	82.31%	91.39%	75.95%	66.34%	0.895 (0.849–0.941)
		Test set	73.89%	92.33%	82.66%	91.67%	77.11%	67.45%	0.892 (0.845–0.939)
V4	XgBoost	Training set	78.01%	77.89%	77.95%	80.51%	77.89%	57.13%	0.879 (0.83–0.928)
		Test set	75.78%	68.67%	72.32%	73.87%	75.64%	46.86%	0.863 (0.811–0.915)
	LR	Training set	56.90%	93.12%	74.34%	90.15%	66.74%	53.34%	0.770 (0.704–0.836)
		Test set	55.56%	93.44%	73.76%	89.83%	66.66%	52.55%	0.756 (0.688–0.824)
	GNB	Training set	57.24%	93.36%	74.64%	90.29%	67.01%	53.84%	0.868 (0.817–0.919)
		Test set	57.56%	91.44%	73.82%	88.36%	66.85%	51.98%	0.864 (0.812–0.916)

## Data Availability

Data will be shared with other researchers by email application for further analysis.

## Supplementary Materials

The following supporting information can be downloaded at: https://www.mdpi.com/article/10.3390/metabo15030205/s1, Table S1: List of indicator changes in serum. Table S2: High-performance liquid chromatography (HPLC) condition for carnitines’ detection. Table S3: Optimized parameters for 76 carnitine indicators (55 carnitines and 21 corresponding ratios) detection using quadrupole trap mass spectrometry. Table S4: Logistic regression analysis of predictive factors for RA.
