# Supplementary material for: Targeted Detection of 76 Carnitine Indicators Combined with a Machine Learning Algorithm Based on HPLC-MS/MS in the Diagnosis of Rheumatoid Arthritis
Source: Metabolites. 2025 Mar 18;15(3):205. doi: 10.3390/metabo15030205 (PMC11944147; doi:10.3390/metabo15030205)
Supplement: Supplementary file 1 [file metabolites-15-00205-s001.zip › metabolites-3523217-supplementary.pdf]

Supplementary Table S1: List of Indicator Changes

Indicators	Mean		P
	RA	Control	
Age (years)	47.44±12.91	47.78±13.40	0.276
Female	76.64%	72.55%	0.178
Blood type	AB (11.21%)	AB (15.69%)	0.059
	A (31.00, 28.97%)	A (26.00, 25.49%)	0.261
	B (28.00, 26.17%)	B (26.00, 25.49%)	0.824
	O (36.00, 33.64%)	O (34.00, 33.33%)	0.924
Height (cm)	160.08±9.35	150.46±29.60	0.000
Weight (Kg)	55.13±9.78	49.01±20.48	0.003
Body mass index (kg/㎡)	20.30±4.97	18.74±6.12	0.266
High Pressure (mmHg)	118.32±12.93	121.39±10.52	0.238
Low Pressure (mmHg)	73.00±6.79	73.60±6.37	0.343
Smoking	8.00 (7.48%)	12.00 (10.76%)	0.114
Drinking	8.00 (7.48%)	6.00 (5.88%)	0.564
Diabetes	0.00	0.00	
Hypertension	8.00 (7.48%)	0.00	0.000
Sports	0.00	30.00 (29.41%)	0.000
C0 (μmol/L)	14.03±4.27	18.2±6.13	0.002
C2 (μmol/L)	3.91±2.06	3.36±1.64	0.166
C3 (μmol/L)	0.32±0.16	0.33±0.2	0.097
C4 (μmol/L)	0.16±0.09	0.3±0.31	0.000
C5 (μmol/L)	0.08±0.08	0.08±0.03	0.077
C6 (μmol/L)	0.13±0.09	0.25±0.28	0.000
C7 (μmol/L)	0.06±0.04	0.05±0.03	0.428
C8 (μmol/L)	0.13±0.09	0.19±0.14	0.026
C9 (μmol/L)	0.02±0.01	0.03±0.01	0.376
C10 (μmol/L)	0.17±0.13	0.25±0.18	0.116
C12 (μmol/L)	0.04±0.02	0.05±0.03	0.092
C14 (μmol/L)	0.03±0.02	0.03±0.01	0.082
C16 (μmol/L)	0.07±0.03	0.07±0.03	0.331
C17 (μmol/L)	0.02±0.01	0.02±0.01	0.478
C18 (μmol/L)	0.04±0.02	0.04±0.02	0.417
C20 (μmol/L)	0.01±0.01	0.01±0.01	0.287
C22 (μmol/L)	0.01±0.01	0.01±0.01	0.596
C24 (μmol/L)	0.02±0.01	0.02±0.01	0.807
C25 (μmol/L)	0.01±0.01	0.01±0.01	0.620
C26 (μmol/L)	0.01±0.01	0.01±0.01	0.173
C5:1 (μmol/L)	0.07±0.06	0.07±0.06	0.452
C8:1 (μmol/L)	0.39±0.19	0.43±0.13	0.001
C10:1 (μmol/L)	0.18±0.1	0.27±0.13	0.047
C10:2 (μmol/L)	0.06±0.04	0.07±0.04	0.635

C10:3 (μmol/L)	0.14±0.09	0.17±0.09	0.522
C12:1 (μmol/L)	0.09±0.03	0.09±0.03	0.161
C14:1 (μmol/L)	0.06±0.03	0.06±0.04	0.805
C14:2 (μmol/L)	0.04±0.03	0.06±0.04	0.138
C16:1 (μmol/L)	0.03±0.01	0.03±0.02	0.453
C18:1 (μmol/L)	0.09±0.03	0.09±0.04	0.005
C18:2 (μmol/L)	0.07±0.03	0.08±0.03	0.015
C20:1 (μmol/L)	0.01±0.01	0.01±0.01	0.316
C20:2 (μmol/L)	0.01±0.01	0.01±0.01	0.399
C20:3 (μmol/L)	0.02±0.01	0.01±0.01	0.005
C3DC (μmol/L)	0.05±0.04	0.05±0.04	0.907
C4DC (μmol/L)	0.05±0.04	0.05±0.03	0.611
C10OH (μmol/L)	0.06±0.04	0.06±0.03	0.867
C6DC (μmol/L)	0.02±0.01	0.02±0.01	0.470
C8DC (μmol/L)	0.02±0.01	0.01±0.01	0.888
C10DC (μmol/L)	0.05±0.02	0.05±0.02	0.973
C12DC (μmol/L)	0.02±0.02	0.02±0.01	0.002
C14DC (μmol/L)	0.01±0.01	0.01±0.01	0.339
C16DC (μmol/L)	0.03±0.02	0.02±0.01	0.000
C18DC (μmol/L)	0.01±0.01	0.01±0.01	0.473
C20DC (μmol/L)	0.01±0.01	0.01±0.01	0.787
C4OH (μmol/L)	0.05±0.05	0.05±0.05	0.213
C5OH (μmol/L)	0.05±0.03	0.04±0.02	0.006
C6OH (μmol/L)	0.04±0.03	0.04±0.03	0.878
C12OH (μmol/L)	0.02±0.01	0.01±0.01	0.108
C14OH (μmol/L)	0.02±0.01	0.02±0.02	0.217
C16:1OH (μmol/L)	0.02±0.01	0.02±0.01	0.485
C16OH (μmol/L)	0.02±0.01	0.01±0.01	0.136
C18:1OH (μmol/L)	0.01±0.01	0.01±0.01	0.508
C18OH (μmol/L)	0.01±0.01	0.01±0.01	0.391
C20OH (μmol/L)	0.01±0.01	0.01±0.01	0.166
C0/C2	6.02±7.33	7.15±4.32	0.261
C0/C16	230.42±103.96	282.53±103.57	0.697
C3/C0	0.02±0.01	0.02±0.02	0.006
C3/C2	0.12±0.1	0.11±0.07	0.019
C3/C16	5.16±2.78	5.53±4.18	0.001
C4/C3	0.53±0.33	0.94±0.88	0.000
C5/C4	0.79±0.82	0.45±0.34	0.002
C5DC/C8	0.48±0.44	0.4±0.29	0.426
C5DC/C16	0.85±0.58	0.97±0.63	0.278
C8/C2	0.04±0.02	0.07±0.06	0.000
C8/C3	0.48±0.4	0.69±0.61	0.001
C8/C10	0.95±0.54	0.81±0.35	0.009
C8/C12	3.65±2.18	4.15±2.19	0.920

C8/C16	2.04±1.27	2.81±1.6	0.178
C14:1/C16	0.93±0.59	0.86±0.37	0.009
C16OH/C16	0.25±0.23	0.21±0.22	0.382
C24/C22	0.73±0.93	0.64±0.8	0.634
C25/C22	0.47±0.59	0.16±0.36	0.000
C26/C20	0.62±0.67	0.59±0.76	0.302
C26/C22	0.59±0.7	0.46±0.69	0.570
C26/C24	0.62±0.7	0.74±0.69	0.804
G (g/L)	30.65±5.21	28.37±2.60	0.000
Glucose (mmol/L)	4.93±0.47	4.88±0.39	0.370
ESR (mm/h)	18.13±14.59	7.26±4.01	0.000
CCP (U/mL)	684.63±601.81	99.66±165.89	0.000
ASO (IU/mL)	62.43±74.23	50.41±30.02	0.120
RF (IU/mL)	92.04±152.75	12.3±11.69	0.000
CRP (mg/L)	3.50±6.13	2.40±3.19	0.010
IgG (g/L)	1385.8±306.01	1309.27±1450.67	0.080
IgA (g/L)	279.58±140.8	252.05±118.85	0.800
IgM (g/L)	138.88±55.38	130.87±75.79	0.010
AKA	0.46±0.46	0.00±0.00	0.000
A/G	1.49±0.30	1.60±0.16	0.000
AFP (ng/mL)	2.91±2.00	3.01±2.26	0.990
AFU (U/L)	17.46±5.05	20.27±6.40	0.420
ALB (g/L)	44.05±3.91	45.02±2.45	0.000
ALP (IU/L)	84.40±30.50	68.34±18.25	0.000
ALT (IU/L)	24.73±29.55	16.88±6.92	0.000
APOA (g/L)	1.01±0.31	1.47±0.15	0.190
APOB (g/L)	0.73±0.26	0.67±0.18	0.460
APTT (s)	23.31±9.33	26.30±2.11	0.340
AST (IU/L)	24.36±13.19	18.44±4.84	0.000
AST/ALT	1.30±0.54	1.21±0.41	0.050
Anti-TPO (IU/mL)	59.82±105.93	33.15±107.16	0.490
BACT (/µL)	1730.91±7801.35	1140.5±5181.39	0.480
BASO (10E9/L)	0.03±0.02	0.03±0.01	0.000
BASO%	0.01±0.00	0.01±0.00	0.000
BU (mmol/L)	5.47±1.80	5.04±1.28	0.110
CA125 (U/mL)	35.45±57.69	10.47±3.57	0.000
CA153 (U/mL)	5.23±3.42	8.35±3.35	0.700
CA199 (U/mL)	10.81±14.92	9.34±5.92	0.080
CAST (µL)	0.38±0.59	0.07±0.16	0.000
CEA (ng/mL)	1.07±0.76	1.60±1.48	0.110
CHO (mmol/L)	4.03±1.41	4.25±0.52	0.000
CK (IU/L)	51.75±39.15	95.88±37.83	0.900
CK-MB (IU/L)	11.54±7.85	10.86±2.04	0.070
CL (mmol/L)	96.33±30.14	103.91±1.52	0.010

CO2 (mmol/L)	22.67±7.67	24.95±1.65	0.020
CRE (μmol/L)	57.45±16.02	63.01±12.68	0.420
Ca (mmol/L)	2.02±0.64	2.22±0.28	0.110
CysC (mg/L)	0.84±0.25	0.83±0.13	0.000
DBIL(μmol/L)	4.29±1.91	4.09±1.07	0.000
EC (μmol/L)	19.22±24.06	6.78±16.65	0.000
EO (10E9/L)	0.12±0.10	0.10±0.07	0.030
EO%	0.02±0.02	0.02±0.01	0.020
ERY	0.21±0.61	0.09±0.45	0.040
FIB (g/L)	3.06±1.62	3.35±0.77	0.350
FT3 (pmol/L)	4.50±1.42	4.58±0.57	0.000
FT4 (pmol/L)	15.66±4.85	15.89±2.22	0.000
GGT (IU/L)	23.34±19.91	18.34±7.34	0.000
HBDH (IU/L)	129.39±41.19	123.29±8.83	0.070
HBeAg (S/CO)	0.14±0.18	0.01±0.05	0.000
HBsAg (S/CO)	139.14±827.4	0.03±0.17	0.010
HCT	0.40±0.04	0.43±0.04	0.090
HDL-C (mmol/L)	1.28±0.47	1.36±0.25	0.000
HFR	0.02±0.02	0.02±0.02	0.630
HGB (g/L)	126.67±14.36	139.92±14.59	0.340
HIV Ag/Ab	0.11±0.05	0.11±0.07	0.380
Hy.CAST (/μL)	0.28±0.44	0.04±0.09	0.000
IBIL(μmol/L)	8.30±4.17	8.25±2.16	0.000
INR	0.86±0.30	0.89±0.04	0.280
K (mmol/L)	3.71±1.21	4.07±0.29	0.050
KET	0.03±0.24	0.00±0.00	0.010
LAP (IU/L)	42.21±16.69	44.20±6.28	0.000
LDH (IU/L)	185.60±64.89	155.00±16.13	0.110
LDH-1 (IU/L)	33.57±15.05	24.00±5.51	0.060
LDL (mmol/L)	2.31±1.10	2.51±0.47	0.000
LEU	0.59±0.91	0.08±0.31	0.000
LFR	0.80±0.29	0.87±0.05	0.080
LH (mIU/mL)	14.61±12.10	6.58±2.21	0.090
LYMPH (10E9/L)	1.66±0.65	1.90±0.47	0.050
LYMPH%	0.29±0.09	0.33±0.07	0.020
Large-RBC	174.20±847.16	1.40±2.08	0.480
MCH (pg)	29.29±2.34	30.16±1.78	0.000
MCHC (g/L)	317.75±11.73	324.21±8.67	0.010
MCV (fl)	127.03±170	93.02±4.10	0.000
MFR	0.07±0.06	0.11±0.03	0.030
MONO (10E9/L)	0.44±0.17	0.39±0.11	0.000
MONO%	0.08±0.02	0.07±0.02	0.050
MPV (fl)	10.64±1.01	11.27±0.89	0.030
MUCUS (/μL)	1.55±3.49	14.04±53.73	0.000

NEUT (10E9/L)	3.70±1.79	3.42±1.03	0.000
NEUT%	0.61±0.10	0.58±0.07	0.010
NIT	0.03±0.18	0.01±0.11	0.040
Na (mmol/L)	129.82±40.55	142.08±1.25	0.010
Non SEC (/μL)	3.87±5.22	0.56±0.63	0.000
Non-Lysed (/μL)	187.88±865.72	4.70±4.78	0.480
Non-Lysed%	63.58±24.54	73.13±17.35	0.530
P-LCR	0.30±0.08	0.35±0.07	0.050
PDW (fl)	12.44±2.32	13.98±2.40	0.390
PH	5.95±0.83	6.13±0.58	0.020
PLT (10E9/L)	230.92±72.96	226.80±54.42	0.020
PRL (ng/mL)	16.64±10.17	10.26±2.93	0.000
POST-ROMA	21.16±19.02	8.70±3.55	0.000
PRE-ROMA	4.45±0.98	6.45±4.34	0.370
PRO	0.30±0.41	0.02±0.15	0.000
PT (s)	9.72±3.39	10.23±0.40	0.280
PT%	90.61±38.28	104.47±6.49	0.180
Path.CAST (/μL)	0.06±0.14	0.03±0.10	0.020
RBC (10E12/L)	4.33±0.45	4.60±0.61	0.340
RDW (fl)	45.92±6.42	43.24±2.47	0.000
RDW%	0.14±0.02	0.13±0.01	0.000
RET (10E12/L)	0.06±0.04	0.07±0.02	0.270
RET%	1.34±0.81	1.57±0.36	0.140
SG	1.02±0.01	1.01±0.01	0.250
SRC	3.17±3.97	1.74±2.97	0.330
SOD (U/mL)	128.00±61.10	169.67±5.69	0.290
Small-RBC	13.63±34.46	3.23±2.76	0.340
Squa.EC (/μL)	14.64±21.69	6.16±16.32	0.010
TBA (μmol/L)	3.53±5.35	2.87±1.64	0.050
T3 (nmol/L)	1.69±0.49	1.74±0.24	0.000
T4 (nmol/L)	100.07±32.08	94.81±13.81	0.000
TBIL (μmol/L)	12.59±5.67	12.34±3.09	0.000
TG (mmol/L)	6.90±19.91	4.43±6.36	0.040
TP (g/L)	74.70±6.06	73.39±3.63	0.000
TSH (μIU/mL)	3.96±5.35	2.46±1.13	0.000
TT (s)	15.44±5.49	16.60±0.40	0.220
UA (μmol/L)	262.61±83.05	296.73±60.29	0.010
UBG	0.54±0.14	0.28±0.26	0.000
VD total (nmol/L)	38.75±22.85	43.74±15.16	0.370
VEGF (pg/ml)	401.85±809.04	60.02±35.74	0.010
VC	0.00±0.00	0.02±0.16	0.530
WBC (10E9/L)	5.95±2.22	5.79±1.36	0.000
WBC Clumps (/μL)	0.49±1.81	0.00±0.03	0.000
X^TAL (/μL)	2.73±17.07	0.43±2.13	0.070

YLC (/µL)	0.91±8.55	0.00±0.00	0.140
aTG (IU/mL)	51.70±100.12	43.41±92.95	0.620
m-AST (IU/L)	8.52±6.71	6.88±2.17	0.010
pANCA	0.25±0.50	0.00±0.00	0.000

Supplementary Table S2: HPLC Condition for 76 carnitine indicators (55 carnitines and 21 corresponding ratios) Detection

Time(min)	Flow Rate(μL/min)	Acetonitrile (%)
0.1	150	100
1	60	100
1.1	500	100
2	500	100
2.1	150	100

Supplementary Table S3: Optimized Parameters for the Detection of 76 carnitine indicators (55 carnitines and 21 corresponding ratios) by Mass Spectrometry on Q-trap

Parameters	Value
CUR	20 psi
CAD	Medium
IS	5500
TEM	450℃
GS1	40 psi
GS2	50 psi
DP	75
EP	10
CXP	3

Supplementary Table S4: Logistic Regression Analysis of Predictive Factors for RA

Variables	P	OR	95% CI	
			Lower Limit	Upper Limit
C2	0.002	1.446	1.151	1.816
C7	0.006	2152115	59.369	7.8E+10
C14	0.019	8.98E+11	90.111	8.95E+21
C22	0.033	1.01E+16	18.539	5.52E+30
C25	0.000	1.21E+26	2.23E+12	6.54E+39
C8DC	0.000	1.13E+23	1.33E+11	9.65E+34
C5DC/C8	0.029	4.239	1.164	15.44
C8/C10	0.020	2.529	1.161	5.511
C14:1/C16	0.002	3.663	1.598	8.396
C16OH/C16	0.017	7.132	1.420	35.826
C25/C22	0.000	3.794	1.848	7.787
C0	0.000	0.793	0.728	0.864
C4	0.000	0.002	0.000	0.022
C6	0.000	0.016	0.002	0.155
C8	0.001	0.001	0.000	0.068
C10	0.001	0.003	0.000	0.085
C24	0.006	0.000	0.000	0.000
C10:1	0.000	0.000	0.000	0.003
C14:2	0.005	0.000	0.000	0.002
C10OH	0.040	0.000	0.000	0.617
C4/C3	0.014	0.430	0.219	0.845
C5DC/C16	0.023	0.460	0.236	0.897
C8/C2	0.000	0.000	0.000	0.000
C8/C16	0.000	0.452	0.322	0.633
